# Placental Malaria: A New Insight into the Pathophysiology

**DOI:** 10.3389/fmed.2017.00117

**Published:** 2017-07-25

**Authors:** Lalita Sharma, Geeta Shukla

**Affiliations:** ^1^Department of Microbiology, Panjab University, Chandigarh, India

**Keywords:** malaria, placenta, pregnancy, low birth weight, intra uterine growth retardation

## Abstract

Malaria in pregnancy poses a great health risk to mother and her fetus and results into complications, such as abortion, still birth, intra uterine growth retardation, and low birth weight. The heavy infiltration of *Plasmodium falciparum*-infected RBCs in the intervillous spaces of placenta seems to be responsible for all the complications observed. Infected RBCs in the placenta cause an inflammatory environment with increase in inflammatory cells and cytokines which is deleterious to the placenta. Increased inflammatory responses in the infected placenta result into oxidative stress that in turn causes oxidative stress-induced placental cell death. Moreover, heat shock proteins that are produced in high concentration in stressed cells to combat the stress have been reported in fewer concentrations in malaria-infected placenta. Pathologies associated with placental malaria seems to be the effect of a change in immune status from antibody-mediated immune response to cell-mediated immune response resulting into excess inflammation, oxidative stress, apoptosis, and decreased heat shock protein expression. However, we also need to study other aspects of pathologies so that better drugs can be designed with new molecular targets.

## Introduction

Malaria has been the most devastating infectious parasitic disease of human kind for centuries. In 2015, an estimated 438,000 malaria deaths around the world have been reported, of which approximately 69% (306,000) were children under 5 years of age. Of all malaria deaths, 90% were reported from African regions and rest were from South-East Asia region and the Eastern Mediterranean region (1). Malaria causes a very high risk to the pregnant woman and her fetus/new born. In malaria endemic areas, it is estimated that at least 25% of pregnant women are infected with malaria, which attribute to more than 20% of all maternal deaths. Malaria accounts for over 10,000 maternal and 200,000 neonatal deaths per year globally (2). Malarial infection in pregnant women results into clinical complications, such as anemia, pulmonary edema, hypoglycemia, cerebral malaria, puerperal sepsis, and some time death too. Consequences of maternal malaria in fetus are abortion, still birth, intra uterine growth retardation (IUGR), premature delivery, and low birth weight (LBW) (3, 4). LBW of the infant has been suspected for poor cognitive and neurosensory development of the child (5–7). WHO recommends that in areas of high malaria transmission, people should be provided with insecticide-treated mosquito nets and intermittent preventive treatment (IPT) with sulphadoxine–pyrimethamine should be given as a part of antenatal care. Although, artemisinin-based combination therapies are highly effective against *P. falciparum* infection, but its use has not been recommended during pregnancy (8). Placenta, which is the interface between mother and fetus, plays important role in successful pregnancy outcome and growth of the fetus that is critically dependent on the placenta. This review summarizes all the pathophysiological processes occurring in the placenta due to malarial infection. The review will give a better insight into understanding placental malaria and help researchers to develop new drug targets.

## Placental Malaria

Malarial infection in placenta is characterized by sequestration of *Plasmodium falciparum*-infected erythrocytes and infiltration of immune cells within the intervillous spaces of the placenta. The placenta turns black due to deposition of the malarial pigment. The parasite densities are much higher in the placenta compared to peripheral blood (9–11). The thickening of placental basement membrane, perivillous fibrinoid deposits, and syncytial knotting results into altered exchange system between mother and fetus. The placental insufficiency to provide nutrients to the fetus causes IUGR (12, 13). The enhanced susceptibility to infections during pregnancy results into high parasitemia and heavy infiltration of parasite-infected RBCs (iRBC) in placental vasculature, a privilege site where the parasite can avoid maternal immune response (14–16). Furthermore, it has been observed that woman who is pregnant for the first time (primigravidae) is more susceptible to malarial infection than woman who has conceived for second or third time (multigravidae). This resistance to the malarial infection in multigravidae is due to the development of placental parasite-specific immunity in second and third pregnancies (17–19).

The severities of malarial infection in pregnant women also depend on prevalence of the infection in a particular community or area. In malaria endemic areas, women with their first pregnancy show more susceptibility to the infection and 20–40% babies born have a LBW. However, multigravidae women develop immunity to placenta-specific *P. falciparum* and are less susceptible to the infection. Contrary to malaria endemic areas, in areas with low malaria incidence, primigravidae and multigravidae are equally susceptible. The more complications of placental malaria in primigravidae are due to the absence of placental parasite-specific immunity which develop in subsequent pregnancies (19–21).

## Molecular Basis of Placental Malaria

The typical and important characteristic of *P. falciparum*-infected erythrocytes is to get attach to vascular endothelium and then sequester to different organs. The erythrocytes, infected with *P. falciparum* express parasite-specific ligands that bind to the receptors on vascular endothelial cells and are responsible for sequestration of infected erythrocytes to organs. The receptors that have been observed on the surface of *P. falciparum*-infected erythrocytes include *P. falciparum* erythrocyte membrane protein 1, 2, and 3 (Pf EMP 1, 2, and 3), histidine-rich protein I and II, sequestrin, rosettins and ring-infected erythrocyte membrane surface antigen (Pf 155/RESA). Ligands to the receptors include intercellular adhesion molecule 1, vascular cell adhesion molecule-1, chondroitin sulfate A (CSA), CD36, TSP, and endothelial leukocyte adhesion molecule-1 (22–24).

*Plasmodium falciparum* isolates from placenta have been found to express var2csa genes that code for Pf EMP1. The Pf EMP1 encoded by var2csa bind to CSA present in placenta and responsible for sequestration. Var2csa gene is over expressed by *P. falciparum* isolates from the placenta (20, 25). Interestingly, *P. falciparum* parasites isolated from placenta do not bind to CD36 that is opposite in nature to *P. falciparum* isolates from non-pregnant women (23, 26). Thus, PfEMP1 proteins encoded by var2csa could prove as potential candidates for vaccine targets (27). Placental isolates of malaria parasite show different degrees of abilities to bind to CSA that may describe the complications of malaria in primigravidae where var2csa show more expression and more binding to CSA (28, 29).

## Immunology of Placental Malaria

The altered physiology and immunity during pregnancy and ability of *P. falciparum*-infected erythrocytes to sequester to various organs are all together responsible for severe malaria in pregnant women especially in first pregnancy and associated IUGR and LBWs in infants (30). The increased concentration of cortisol, during pregnancy makes women more susceptible to malaria by directly inhibiting NK cell activity against *P. falciparum*-infected erythrocytes. In pregnant women cell-mediated immunity (CMI) is halted which is required to support the development of placenta and the fetus. But this halt in CMI make pregnant women more susceptible to intracellular pathogens. However, in malaria-infected pregnant women, an increase in CMI has been observed in the local placental environment. Elevated levels of pro-inflammatory cytokines, such as IFN-γ, IL-2, and TNF-α, in the placenta of malaria-infected women especially in primigravidae account for the observed placental pathology and adverse pregnancy outcomes (31–33). However, IFN-γ levels have also been found elevated in placenta of multigravidae and this scenario can be explained on the bases of development of placental parasite-specific immunity in second or subsequent pregnancies which neutralizes the adverse effect of IFN-γ (34–36).

The levels of chemokines have been observed elevated in the placental intervillous spaces, which correlate with increased monocyte density, parasite density, and malaria pigments in the placenta. Maternal macrophages are the predominant source of chemokines in the placenta, but fetus cells can also contribute. The chemokines help to recruit macrophages, cytotoxic T cells, B cells, and granulocytes in the placenta and contribute to the pathologies of placental malaria. There is excessive sequestration of iRBC and leukocytes in the intervillous spaces of placenta and formation of perivillous fibrin clot during malarial infection, which interfere blood flow across the placenta thus, nutrients to the fetus (37, 38). The low levels of HDL-C and increased TG levels in plasma of malaria-infected pregnant women may also be one of the factors for adverse pregnancy outcomes in malaria (39). Placental pathology in malaria is caused by expression of unique Pf EMP1 protein on iRBC which helps the parasite to sequester into the placenta. Women, who have malaria during pregnancy, develop Pf EMP1-specific antibodies and these antibodies protect women from malaria in subsequent pregnancies (40–43).

## Reactive Oxygen Species (ROS) and Placental Malaria

A balance between ROS and antioxidants is required for normal physiology in animals and any alteration that results in overabundance of ROS cause stress and damage to cells, tissues, and organs (44, 45). Several studies have documented increased oxidative stress during pregnancy, affecting the multiple physiological processes, i.e., oocyte maturation and fertilization, embryo development, initiation of preterm labor, and normal parturition (46–48). The antigenic stimulation due to malarial infection activates the macrophages and activated macrophages release ROS to destroy the intracellular parasite (49, 50). ROS are also produced by the *Plasmodium* parasite for degradation of hemoglobin (51, 52). To some extent, ROS are beneficial; by helping the patient to fight intra cellular parasite. But massive production of ROS by infiltered immune cells in the placenta results into damage of vascular lining and placental cells (53–55). A high rate of lipidperoxidation has been detected in the placenta of mouse infected with *P. berghei* where lipidperoxidation increased as the infection progressed. The same study also reported low catalase activity and low levels of SOD and GSH in the placenta and suggested that it might be responsible for high lipidperoxidation in the placenta (56–58). However, there are many other studies that suggest that reduced antioxidant levels are not responsible for oxidative stress in the placenta in malarial infection (59). Moreover, ROS generation by immune cells and infected erythrocytes also add to the oxidative stress in *Plasmodium* parasite-infected placenta. The increase in lipid peroxidation in the placenta of malaria-infected pregnant women could be responsible for increase in the serum triglycerides, cholesterol, and low density lipoproteins in malaria-positive primigravidae (60). In cerebral malaria, ROS generation by monocytes and neutrophils at blood–brain barrier has been found to be responsible for damage to vascular cell lining and related complications (61–63). Thus, it seems that infiltration of *P. falciparum*-infected erythrocytes and immune cells to intervillous spaces of placenta results into production of ROS, increased TNF-α concentration and oxidative stress that contribute to placental pathology and associated clinical complications.

## Apoptosis and Placental Malaria

Apoptosis is a programmed cell death which helps in maintaining homeostasis in the body. Impaired regulation of apoptosis has been found to be responsible for etiology of many diseases, e.g., cancer and autoimmune diseases (64, 65). On the other side, excessive death of cells due to apoptosis has been associated with neurodegenerative diseases and tissue damage in many diseases (66–68). A recent study has reported extensive cell death in the placenta of *P. berghei*-infected mice that have been linked to oxidative stress-induced apoptosis in the placenta (56). From the study, it is quite evident that oxidative stress and apoptosis are the two main reasons for observed placental pathology and related adverse effects in malaria-infected women and infants. However, a study has shown the effectiveness of sulphadoxine–pyrimethamine, the drug used for IPT in pregnancy and chloroquine in reducing pathologies of placenta induced by oxidative stress and apoptosis (69).

## Heat Shock Proteins (HSPs) and Placental Malaria

Heat shock proteins as suggested by their name are produced by cells in large quantities after a shock or a stress signal. These proteins help cells to recover from an injury or a shock (70). A wide range of HSPs have been observed throughout the pregnancy and found to be important for normal morphology and physiology of the placenta (71–73). HSPs are among the earliest proteins produced during zygote formation and are important for development of the embryo (74–76). In the last decade, there have been many articles stating the importance of HSPs in defense mechanisms against infectious as well as non-infectious diseases (77, 78). The role of HSPs in inhibition of apoptosis has been documented and found to be associated with cancer development. Many mechanisms of apoptosis inhibition have been reported, such as binding of HSP 90 to caspase-3 and HSP 60 to Bax and Bak proteins (79–81). However, Hsp 70 interferes with binding of Apaf-1 to procaspase-9 (82, 83). HSP 25 is also known for its anti-apoptotic properties and plays important role in embryonic development (84, 85).

Malarial infection during pregnancy has been reported to cause HSPs response in placenta especially HSP 90, 70, 60, and 25 were detected at high levels in the placenta of mouse model of malaria infection. However, the levels of HSPs 70, 60, and 25 in the placenta decreased with progression of malarial infection. On the other hand, HSP 90 response increased gradually with increase in parasite densities in the placenta. The decrease in HSPs 70, 60, and 25 levels seems to be responsible for massive cell death in the placenta of *P. berghei*-infected mice and suggest the inefficiency of HSP 90 alone to repair the cell damage (56, 86). This study demonstrated that HSPs and cell damage in the malaria-infected placenta are inversely proportion to each other. Therefore, high HSP levels in malaria-infected placenta are important to maintain the normal placental architecture and physiology. Based on the study, therapies can be sought out which can increase the HSP levels in malaria-infected placenta.

## Conclusion

**Figure 1:**
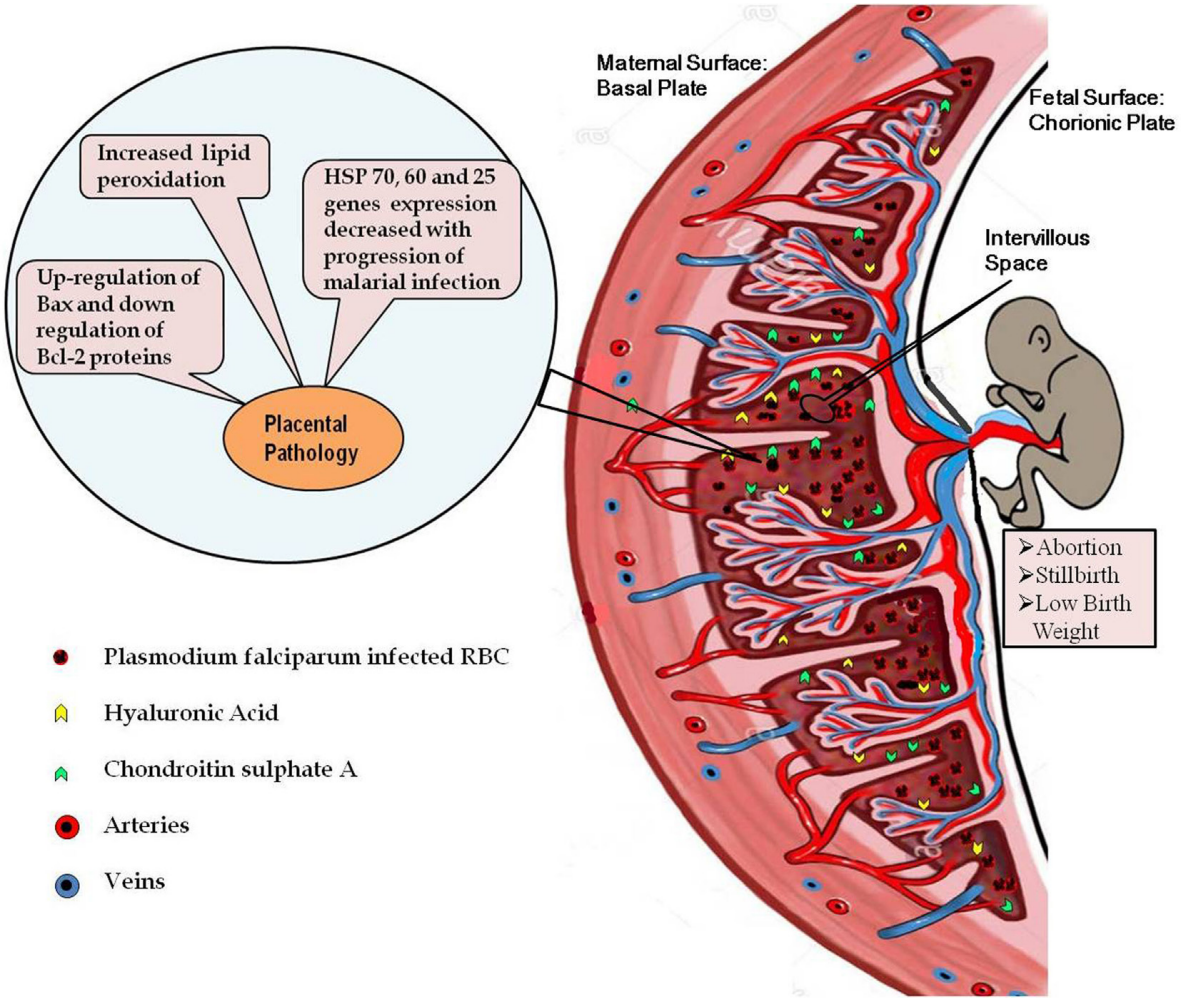
Diagrammatic representation of the placental malaria and implications. Malarial infection during pregnancy results into infiltration of the parasite-infected RBCs to the intervillous space of placenta resulting into exacerbated inflammatory response. High inflammation causes oxidative stress-induced apoptotic cell death in the placenta. Decreased expressions of the heat shock protein genes in the infected placenta further contribute to the placental pathology. All these pathological alterations in the placenta contribute to the poor pregnancy outcomes associated with malarial infection.

Malaria infection during pregnancy is substantial health risk for the pregnant woman as well as to her fetus and newborn. The general immune suppression during pregnancy makes women more susceptible to many infections, including malarial infection especially during first trimester. During pregnancy, cell-mediated immune response is very low that is required to sustain the placenta, a new organ in first pregnancy. However, low cell-mediated immune response in placenta makes it preferred site for the parasite to hide from host immune responses. However, as malarial infection progresses, there is an increase in CMI response resulting in massive recruitment of macrophages to intervillous spaces of placenta and increased concentrations of TNF-α and IFN-γ to counteract the parasite iRBC. But this increase in CMI response is deleterious to the placenta which causes oxidative stress and apoptotic cell death in placenta, leading to poor pregnancy outcome, such as abortions, still birth, IUGR, and LBW. Moreover, HSPs that are important for maintenance of normal morphology and physiology of the placenta have been reported in low quantities in malaria-infected placenta. The low HSP levels contribute indirectly to the observed placental pathology by not able to control the cell damage caused by malarial infection. Therefore, placental cell damage and associated consequences in fetus and newborn are accumulative effect of exacerbated inflammation, oxidative stress, apoptosis, and low levels of HSPs in the infected placenta (Figure 1). However, we also need to study other aspects of placental pathologies, especially molecular pathways governing immune responses so that therapies can be designed to modulate the immune response in favor of the host.

## Author Contributions

LS: literature survey and wrote the article. GS: edited the article.

## Conflict of Interest Statement

The authors declare that the research was conducted in the absence of any commercial or financial relationships that could be construed as a potential conflict of interest. The handling editor and the reviewer ML declared their shared affiliation, and the handling editor states that the process nevertheless met the standards of a fair and objective review.
